# The genomic landscape of undifferentiated embryonal sarcoma of the liver is typified by C19MC structural rearrangement and overexpression combined with *TP53* mutation or loss

**DOI:** 10.1371/journal.pgen.1008642

**Published:** 2020-04-20

**Authors:** Bhuvana A. Setty, Goodwin G. Jinesh, Michael Arnold, Fredrik Pettersson, Chia-Ho Cheng, Ling Cen, Sean J. Yoder, Jamie K. Teer, Elsa R. Flores, Damon R. Reed, Andrew S. Brohl

**Affiliations:** 1 Division of Hematology/Oncology/BMT, Nationwide Children’s Hospital, Columbus, Ohio, United States of America; 2 Department of Pediatrics, The Ohio State University Wexner Medical Center Columbus, Ohio, United States of America; 3 Chemical Biology and Molecular Medicine Program, Moffitt Cancer Center, Florida, United States of America; 4 Department of Pathology and Laboratory Medicine, Nationwide Children's Hospital, Columbus, Ohio, United States of America; 5 Department of Pathology, The Ohio State University Wexner Medical Center, Columbus, Ohio, United States of America; 6 Department of Biostatistics and Bioinformatics, Moffitt Cancer Center, Tampa, Florida, United States of America; 7 Molecular Genomics Core Facility, Moffitt Cancer Center, Tampa, Florida, United States of America; 8 Department of Molecular Oncology, Moffitt Cancer Center, Tampa, Florida, United States of America; 9 Adolescent and Young Adult Program, Moffitt Cancer Center, Tampa, Florida, United States of America; 10 Sarcoma Department, Moffitt Cancer Center, Tampa, Florida, United States of America; Hopp-Children's Cancer Center KiTZ and German Cancer Research Center DKFZ, GERMANY

## Abstract

Undifferentiated embryonal sarcoma of the liver (UESL) is a rare and aggressive malignancy. Though the molecular underpinnings of this cancer have been largely unexplored, recurrent chromosomal breakpoints affecting a noncoding region on chr19q13, which includes the chromosome 19 microRNA cluster (C19MC), have been reported in several cases. We performed comprehensive molecular profiling on samples from 14 patients diagnosed with UESL. Congruent with prior reports, we identified structural variants in chr19q13 in 10 of 13 evaluable tumors. From whole transcriptome sequencing, we observed striking expressional activity of the entire C19MC region. Concordantly, in 7 of 7 samples undergoing miRNAseq, we observed hyperexpression of the miRNAs within this cluster to levels >100 fold compared to matched normal tissue or a non-C19MC amplified cancer cell line. Concurrent *TP53* mutation or copy number loss was identified in all evaluable tumors with evidence of C19MC overexpression. We find that C19MC miRNAs exhibit significant negative correlation to TP53 regulatory miRNAs and K-Ras regulatory miRNAs. Using RNA-seq we identified that pathways relevant to cellular differentiation as well as mRNA translation machinery are transcriptionally enriched in UESL. In summary, utilizing a combination of next-generation sequencing and high-density arrays we identify the combination of C19MC hyperexpression via chromosomal structural event with *TP53* mutation or loss as highly recurrent genomic features of UESL.

## Author summary

We perform the most comprehensive molecular analysis to date of undifferentiated embryonal sarcoma of the liver, utilizing whole exome sequencing, RNA sequencing, miRNA sequencing, and SNP arrays. We find that UESL tumors harbor aberrant transcriptional start sites within C19MC region that is driven by structural rearrangement, leading to extreme overexpression of C19MC miRNAs. We further find that C19MC miRNAs negatively correlate with TP53 and K-Ras regulatory miRNAs. UESL also harbors highly recurrent *TP53* mutation or copy number loss. Therefore, we provide for the first time a genomic landscape of UESL and describe cellular consequences of the discovered foundational genomic changes.

## Introduction

Undifferentiated embryonal sarcoma of the liver (UESL) is an aggressive primitive malignancy. It occurs predominantly in children with a peak age incidence of 6–10 years and equally across gender [1]. UESL mostly arises from the right lobe of the liver [2], presenting with nonspecific symptoms of abdominal pain, fever, nausea and anorexia. Serum α-fetoprotein is usually normal[3]. Current treatment strategies include multimodal approaches with tumor resection and adjuvant chemotherapy, with mortality considered to be primarily from recurrent or metastatic disease[4]. The prognosis for patients with recurrent or metastatic disease is poor. No disease-specific or molecularly guided therapies have been currently reported.

Though the oncogenesis of UESL remains uncertain, in some cases UESL can arise from malignant transformation of mesenchymal hamartoma of the liver (MHL) [5–7]. Supporting the hypothesis that these two tumors are related, conventional cytogenetic studies as well as targeted sequencing approaches have identified recurrent chromosomal alterations affecting a noncoding region on chr19q13.4 in several cases of UESL as well as recurrently in mesenchymal hamartoma [8]. The 19q13.4 locus, previously termed MHLB1 on account of the association with MHL, is a gene-poor area with highly repetitive sequence. A variety of inter- and intra- chromosomal translocation partners to 19q13.4 have been described in UESL and MHL, with t(11;19)(q11;q13.4) linking *MALAT1* to 19q13.4 being the most recurrently reported [8]. Given the noncoding nature and lack of an obvious oncogene in the vicinity of 19q13.4, disruption of a regulatory region of either a neighboring gene or of the nearby chromosomal 19 microRNA cluster (C19MC) have been hypothesized as a catalyst for tumorigenesis.

The chromosome 19 miRNA cluster has recently gained interest as a potential oncomir. C19MC has been implicated in various tumors including but not limited to embryonal tumors with multilayered rosettes (a pediatric tumor type affecting central nervous system) [9], breast cancer[10], hepatocellular carcinoma[11], parathyroid tumors[12], infantile hemangioma[13], testicular germ cell tumors[14], and thyroid adenomas[15]. C19MC is the largest human microRNA cluster, spanning 96kb and containing 46 individual miRNAs [16]. In adult tissues, C19MC miRNAs are only expressed in the placenta [16], and therefore at least theoretically make an attractive target for therapeutic intervention. In the placenta, C19MC plays a pivotal role in trophoblast differentiation and migration as well as in providing resistance to viral infection [17].

The primary objective of our study was to perform a survey of the genomic landscape of UESL to determine the most common recurrent molecular alterations. Using patient samples, we perform a combination of whole exome sequencing, RNA sequencing, miRNA sequencing, and single nucleotide polymorphism (SNP) arrays to evaluate for recurrent mutations, copy number changes, and expressional events. We find that UESL display a highly aneuploid genome with recurrent structural alterations of chr19q13 that are uniformly associated with aberrantly high levels of transcriptional activity of the chromosome 19 microRNA cluster. In addition we find that *TP53* mutation or loss is present in all samples that also display C19MC changes. In light of previous literature and the highly recurrent nature of these events, our study provides compelling evidence that these two genetic events are foundational and perhaps pathognomonic of this disease.

## Results

Fourteen subjects with a diagnosis of undifferentiated embryonal sarcoma of the liver were identified including 5 males and 9 female with median age of 8 years at diagnosis (range 8mo– 11 years). Sequencing studies were performed where quality and quantity of nucleic acids allowed and included either tumor-normal matched (7) or tumor only (7) whole exome sequencing, SNP arrays (13), RNAseq (13) and miRNAseq (7). Additional demographic and sequencing details as well as summary findings are provided in Table 1.

**Table 1:** Patient demographics and summary findings.

COG ID	Study	Studies performed	C19MC finding(s)	TP53 finding(s)	Race	Ethnicity	Sex	Age at diagnosis (days)	Primary tumor size (cm)
PAPWGG	D9902	tumor WES, RNAseq, SNParray, miRNAseq	CNV and overexpression	R273G	Asian	Not Hispanic or Latino	Female	3542	15
PARCGJ	ARST0332	matched WES, RNAseq, SNParray, miRNAseq	CNV and overexpression	R273H	White	Not Hispanic or Latino	Male	1590	11.4
PATJSC	ARST0332	tumor WES, RNAseq, SNParray, miRNAseq	CNV and overexpression	del 190–193	White	Not Hispanic or Latino	Female	3112	11.4
PATTAT	D9902	tumor WES, RNAseq, miRNAseq	overexpression (CNV n/a)	no mutation detected (CNV n/a)	White	Not Hispanic or Latino	Female	3307	11.3
PATWFW	ARST0332	matched WES, RNAseq, SNParray	CNV and overexpression	R248W	Unknown or not reported	Hispanic or Latino	Female	4294	14.2
PATWXD	ARST0332	tumor WES, RNAseq, SNParray, miRNAseq	overexpression	L93fs	White	Not Hispanic or Latino	Female	4236	12
PATJXY	ARST0332	tumor WES, RNAseq, SNParray, miRNAseq	overexpression	del 201–202	Black or African American	Not Hispanic or Latino	Male	1956	13
PAUGAD	ARST0332	tumor WES, SNParray	not evaluable	no mutation detected	White	Hispanic or Latino	Male	4257	13
PAUPDM	ARST0332	matched WES, RNAseq, SNParray	CNV and overexpression	CN loss (chr17:7500764–7627453)	White	Not Hispanic or Latino	Female	2520	17.5
PAUPRI	ARST0332	matched WES, RNAseq, SNParray	CNV and overexpression	R342X	White	Not Hispanic or Latino	Male	3867	15
PAVZCN	D9902	matched WES, RNAseq, SNParray	CNV and overexpression	CN loss (chr17:65310–15491532)	White	Not Hispanic or Latino	Female	3652	13.7
PAWSCY	D9902	matched WES, RNAseq, SNParray	CNV and overexpression	Q136Q	Black or African American	Not Hispanic or Latino	Male	1825	11.6
PAWSXX	D9902	matched WES, RNAseq, SNParray	CNV and overexpression	TR155del	White	Not Hispanic or Latino	Female	272	17.6
PAWYLP	D9902	tumor WES, RNAseq, SNParray, miRNAseq	CNV and overexpression	T125T	White	Not Hispanic or Latino	Female	3065	17

Whole exome sequencing generated a median 136 Million (M) total read pairs, resulting in 107x coverage (range 67x-200x) across the capture region after duplication removal and mapping. RNA sequencing was performed with an average yield of 82M read pairs (range 58-97M). RNAseq data from the one tumor taken from FFPE materials failed quality control and was therefore neither utilized for normalization nor for clustering analysis. miRNAseq was performed to a median of 17M total reads (range 8-22M).

### UESL display significant aneuploidy with recurrent 19q13.42 breakpoints

**Fig 1:**
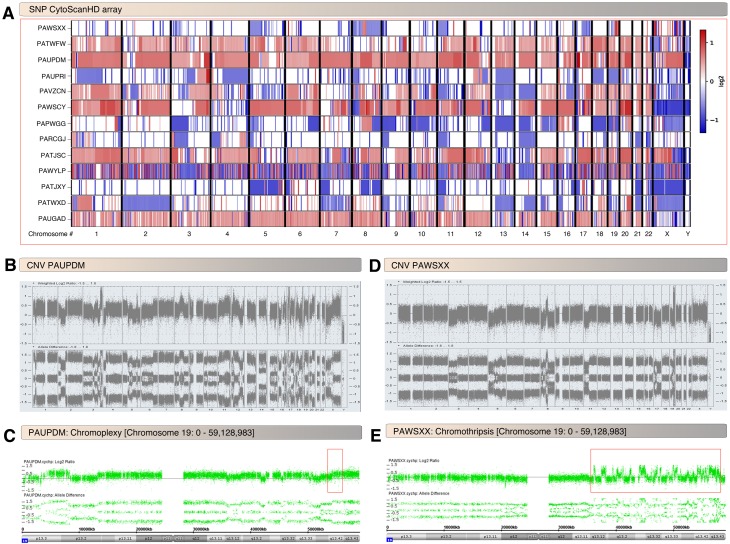
UESL display structural genomic instability. A, Overview of genome wide copy number variation (CNV) in UESL tumors derived from high density SNP arrays. The majority of UESL (top) display frequent CNV indicative of aneuoploidy. A minority of tumors (bottom) displays quiet genomes. B-C, Copy number variations in PAUPDM UESL sample. Frequent copy number change points consistent with a chromoplexy pattern are noted genome-wide (B), and particularly in chromosome 19 (C). Note that there is a copy number change point in chromosome 19 band q13.42 (box). D-E, Copy number variations in PAWSXX UESL sample. This sample displays focal genomic areas of very high frequency CNV changes oscillating between two copy number states suggestive of chromothripsis (D). There is an area of chromothripsis on the long arm of chromosome 19 (box) including at band q13.42 (E).

From SNP array analysis, all 13 analyzed tumors displayed substantial degrees of aneuploidy typified by areas of complex genomic rearrangements suggestive of chromoanagenesis [18] events such as chromothripsis (a phenomenon where multiple double stranded DNA breaks occur in a limited number of chromosomal segments followed by random reassembly leads to high frequency structural variants in isolated genomic regions [19]) or chromoplexy (chromosomal restructuring phenomenon characterized by chained inter- and intra- chromosomal translations with frequent deletions at the breakpoints [20]) (Figs 1 and S1 Fig). The most typical pattern was copy number change points distributed across a large percentage of chromosomes, suggestive of chromoplexy (1B and 1C and S1 Fig). In one tumor, focal high-frequency copy number changes were more regionally isolated to several chromosomal areas and therefore more suggestive of chromothripsis (Fig 1D and 1E and S1 Fig). In 10 of the 13 tumors with chromosomal complexity, there was clear evidence of a copy number change point in 19q34.42, suggestive of unbalanced structural alterations and concordant with previous reports of recurrent chromosomal breaks in this region [8]. Of note, this technology would not be expected to identify chromosomal breaks that do not result in copy number variation, for example a balanced translocation. Notably, the C19MC region had increased copy number in multiple samples (Fig 1C and 1E and S1 Fig). Taken together, these results show that most UESL display significant genomic instability with frequent change points of copy number status in 19q13.42 indicative of chromosomal breakpoints in this region.

### UESL display striking overexpression of C19MC

**Fig 2:**
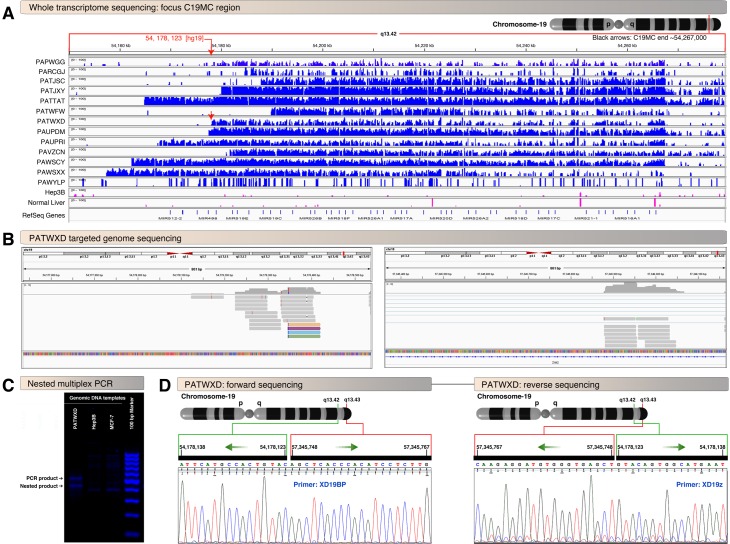
UESL display aberrant transcriptional activity of the C19MC region. **A novel *PEG3/ZIM2*-C19MC fusion is identified.** A, Read counts of mapped whole transcriptome show high levels of aberrant transcriptional activity in the C19MC region. Note that the abrupt starting location of transcriptional mapping is in different co-ordinates in different UESL samples, suggestive of sample specific fusional events. The genomic position of the experimentally verified fusion in PATWXD is indicated by the red arrow in panel-A and notably corresponds to the start (5’ end) of transcriptional activity in this sample. Hep3B cell line (hepatocellular carcinoma) and normal liver samples are shown at the bottom for comparison and as expected show negligible amounts of RNA mapping to this non-coding region. B, Targeted DNA sequencing of PATWXD UESL tumor showing abrupt end of read mapping near the C19MC start site (left) as well as in the *PEG2/ZIM2* gene locus (right, shared gene region). The reads also mark the position of primers designed for gDNA PCR (one primer at 5’ end of reads and another at 3’end of reads for each locus (therefore one set of primers will form a nested primer set). C, Nested multiplex PCR of PATWXD genomic DNA showing amplicon (~550 bp) including the nested product (~450 bp). D, Paired end Sanger sequencing of ~550 bp product from panel-B showing the *PEG3/ZIM2* locus fused to C19MC aberrant transcriptional start site.

Given the evidence of recurrent structural alterations in the vicinity of the chromosome 19 microRNA cluster as well as evidence in another tumor type that translocations to this area can lead to significant C19MC overexpression, we manually inspected mapped whole transcriptome sequencing in this region. Similar to observations in embryonal tumors with multilayer rosettes (EMTR) affected by C19MC fusion [9], we noted striking transcriptional activity across the entire C19MC region (Fig 2A). All 13 UESL tumors with RNAseq data displayed this finding. All samples (10 of 10) with copy number change points in 19q13.42 displayed this C19MC transcriptional activity starting at or near the position of the predicted region of copy number transition. Notably, C19MC transcriptional activity had an abrupt, sample specific start site, suggestive of aberrant expression due to chromosomal structural event disrupting the natural transcriptional start site (Fig 2A).

### Identification of a novel PEG3/ZIM2 locus breakpoint fused to C19MC

Previous reports have shown recurrent fusions involving the C19MC locus in several cases of UESL [8]. It is possible that the aberrant transcriptional activities within C19MC observed (Fig 2A) could be driven by chromosomal structural rearrangements. To attempt to further characterize the chromosomal structural rearrangements involving C19MC in our cohort, we performed targeted sequencing of the upstream C19MC area. In one tumor (PATWXD), manual inspection identified abrupt transition in reads of sequence mapping in the C19MC locus (Chr19q13.42) with more abrupt paired reads mapping within Chr19q13.43 (Fig 2B). Primers and nested primers were designed to span the predicted fusion breakpoint and yielded amplicons in PATWXD sample but not in Hep3B or MCF7 cancer cell lines (Fig 2C). Sanger sequencing of the product confirmed a fusion between *ZIM2/PEG3* and C19MC, Chr19:54,178,123::Chr19:57,345,748 (Hg19 genome build) (Fig 2D). Strikingly, the fusion point co-ordinate Chr19:54,178,123 is at the aberrant transcription start site of PATWXD sample (Fig 2A) suggesting that the fusion with *PEG3/ZIM2* likely drives the aberrant transcription of C19MC in this sample. This is a novel breakpoint but located close to the previously reported C19MC to *PEG3* fusion [8]. The directionality of the fusion event suggests that this is an intrachromosomal inversion event (Fig 2D). For the remaining tumors in our cohort, ambiguous mapping precluded definitive fusion detection despite the use of multiple bioinformatics fusion prediction tools (see Materials and Methods) as well as manual inspection.

### UESL tumors exhibit hyperexpression of individual C19MC miRNAs and display 3p or 5p dominant mature miRNAs

**Fig 3:**
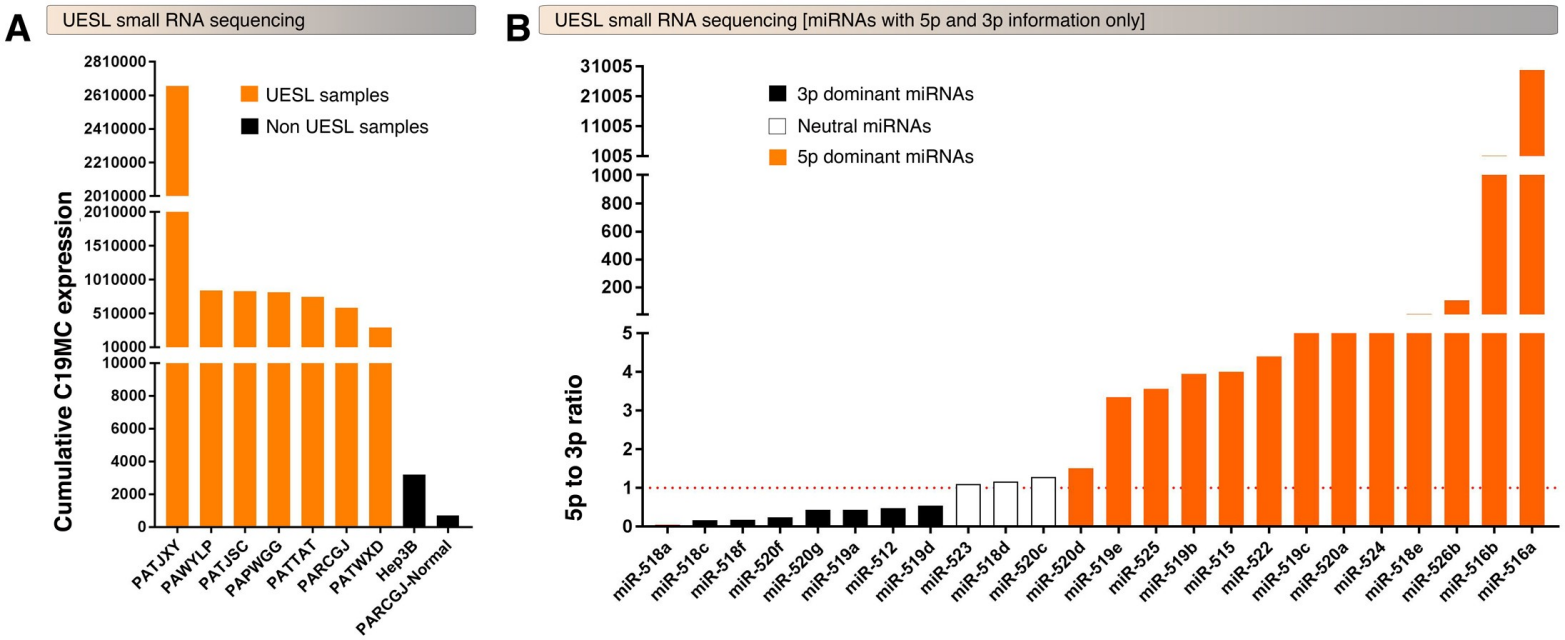
UESL tumors hyperexpress C19MC miRNAs with selective upregulation of 5p or 3p mature miRNAs. A, Small RNA-seq showing overexpression of C19MC miRNAs in UESL tumors compared to normal liver sample or Hep3B cell line. The bar graphs represent the cumulative expression of all 46 C19MC miRNAs (5p and 3p). B, 24 C19MC miRNAs that have 5p and 3p mature miRNA information in UESL tumors display miRNA-specific selective 5p vs. 3p expressional patterns.

Seven of the UESL tumors with sufficient materials underwent small RNA sequencing as well as an adjacent normal liver tissue from one patient and a Hepatocellular carcinoma cell line for comparison. All seven of the evaluated UESL tumors displayed substantial overexpression of the C19MC microRNAs to levels 100–10,000 fold higher than observed in either of the comparators (Fig 3A). This level of C19MC miRNA expression is particularly impressive considering that 1) C19MC miRNAs are amongst the most highly expressed of all miRNAs in UESL tumors (twelve C19MC miRNAs rank in top 50 for average expression) and 2) normal tissues outside of the placenta are expected to have near-zero expression of C19MC miRNAs[16].

Among the 46 individual C19MC miRNAs, 24 had information of both mature 3p and 5p miRNA expression levels and were classified based on the dominant expression of 3p or 5p miRNAs. Eight miRNAs (miR-518a, miR-518c, miR-518f, miR-520f, miR-520g, miR-519a, miR-512, and miR-519d) had dominant expression of 3p over 5p, thirteen miRNAs (miR-520d, miR-519e, miR-525, miR-519b, miR-515, miR-522, miR-519c, miR-520a, miR-524, miR-518e, miR-526b, miR-516b, and miR-516a) had dominant expression of 5p over 3p, and three miRNAs (miR-523, miR-518d, and miR-520c) had nearly equal amount of 5p and 3p mature miRNAs (Fig 3B). Notably, miR-526b, miR-516b, and miR-516a had >100 fold expression of 5p over 3p suggesting that these 5p super dominant miRNAs might sponge corresponding 3p miRNAs. Therefore, not only hyper-expression of C19MC miRNAs but also the relative stability of 3p versus 5p mature forms also might contribute to UESL.

### UESL display positively correlated expression of C19MC miRNA clusters with negative correlation to *TP53/KRAS*-regulatory miRNAs

**Fig 4:**
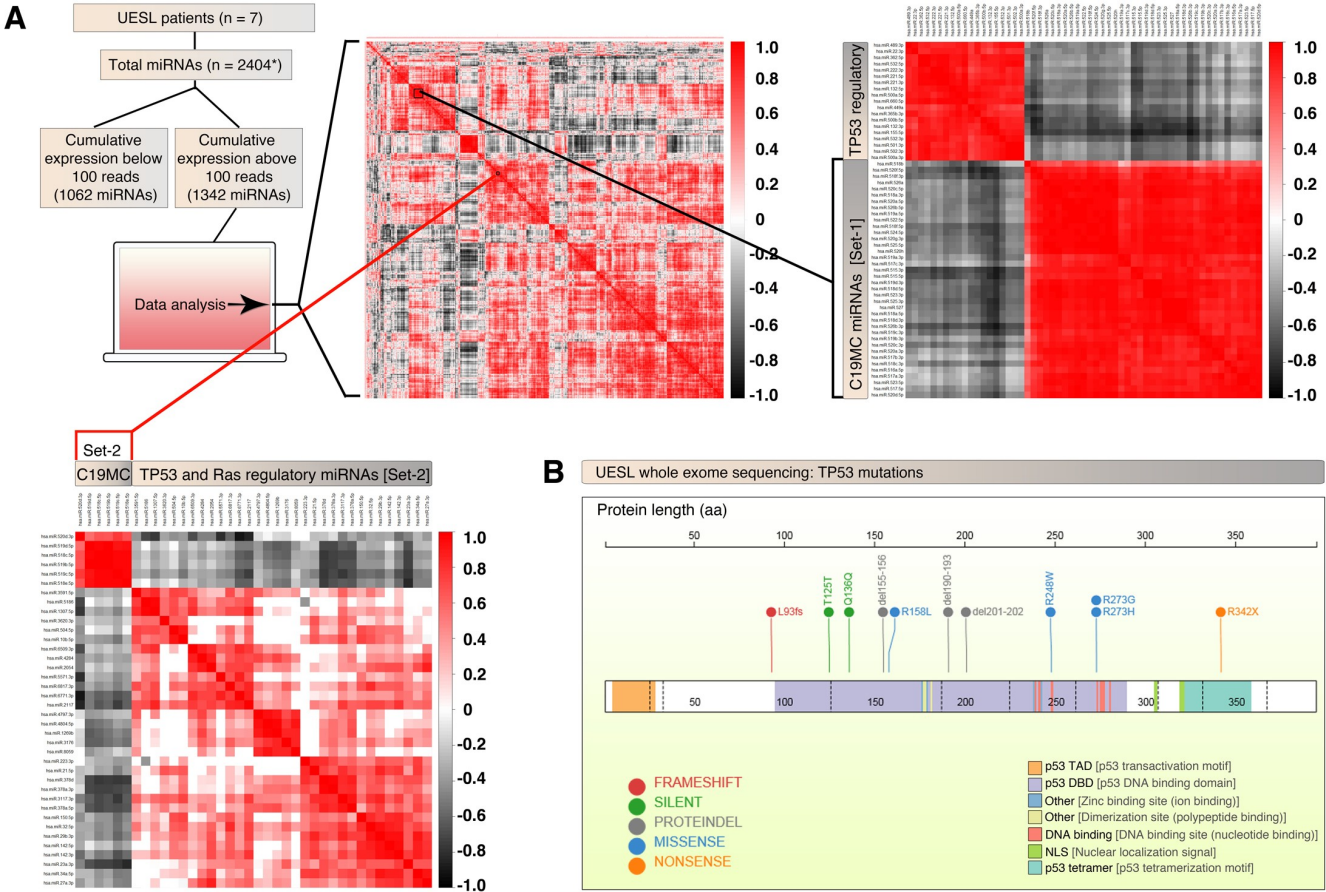
UESL display frequent TP53 mutations and exhibit negative correlation of C19MC miRNAs to TP53 and KRAS-regulatory miRNAs. A, Small RNA-seq of UESL samples were subjected to C19MC miRNA correlation analysis with miRNA transcriptome of 1342 miRNAs (that had a cumulative reads of 100 or more) that were selected from a total of 2404 miRNAs (counting 5p and 3p separately). C19MC miRNAs fall into two clusters (top left heatmap): a large cluster which is negatively correlated to many *TP53* regulatory miRNAs (top right heatmap) and a smaller cluster which is negatively correlated to both *TP53* and *KRAS* regulatory miRNAs (bottom left heatmap). B, UESL harbor an assortment of TP53 mutations as evaluated by whole exome sequencing. Notably, almost all mutations are truncation or known oncogenic missense mutations.

To further understand the expression and function of C19MC miRNAs in the context of other miRNAs, we performed correlation analysis of all expressed miRNAs in the 7 sequenced UESL samples. In UESL, the majority of the C19MC microRNAs (set-1) formed a tightly correlated expression pattern, suggesting shared expressional regulation of the majority of the cluster (Fig 4A and S1 Table). Notably, this tightly correlated cluster of C19MC miRNAs were strongly negatively correlated to a second cluster of TP53-regulatory miRNAs of potential oncologic importance (Fig 4A and S1 Table). Interestingly, many of these negatively correlated miRNAs are directly or indirectly (through K-Ras) related to p53 negative regulation or function [21–28].

Additionally, a smaller subset of C19MC miRNAs (Set-2) formed a distinct correlatively co-expressed cluster, which was negatively correlated to a separate large set of TP53 and KRAS-regulatory miRNAs (Set-2) (Fig 4A and S1 Table). Micro-RNAs from this negatively regulated set have well described K-Ras regulatory [29–33] and or TP53-regulatory function [34–39].

In summary, C19MC microRNAs are substantially overexpressed in UESL and exhibit a strong negative correlation to K-Ras and TP53 regulatory miRNAs.

### UESL display frequent mutation or copy number loss of *TP53*

To elucidate the mutational landscape, we subjected the UESL samples for whole exome sequencing. From the paired whole exome sequencing cohort (n = 7), we observed a median of 39 somatic coding mutations per sample (range: 21–77), placing UES on the low end of the mutational burden spectrum across cancer types, similar to other pediatric malignancies. Only a single gene, *TP53*, was statistically recurrently mutated with somatic mutation identified in 5 of 7 patients with matched sequencing data (Fig 4B). To further evaluate the spectrum of *TP53* alteration in UESL, we additionally evaluated tumor-only sequencing for *TP53* mutation and evaluated for copy number loss of *TP53* from SNP array data. In total, 12 of 13 patients (92.3%) with both mutational and CNV data harbored either *TP53* mutation or copy number loss (Fig 4B). One additional sample lacked *TP53* mutation by exome sequencing but did not have SNP array data for which to evaluate copy number loss. Of note, the one tumor that both lacked *TP53* mutation by exome sequencing and copy number loss by SNP array was not evaluable for C19MC overexpression due to lack of RNA materials for RNA and miRNA sequencing. Notably all tumors with documented C19MC overexpression that were evaluable for both mutation and CNV also harbored *TP53* mutation or loss (12 of 12). From the matched sequencing cohort, we noted several additional possibly pathogenic somatic mutations including singleton mutations in 3 different JAK-STAT pathway genes, though none other than *TP53* that were recurrent at the gene level (S2 Table). Given recent reports of an association of DICER1 syndrome with mesenchymal hamartoma of the liver [40], we specifically analyzed somatic and germline sequencing data for evidence inactivating mutation in *DICER1* but found none in our cohort.

### UESL display enriched transcription of cellular differentiation and viral response gene sets

**Fig 5:**
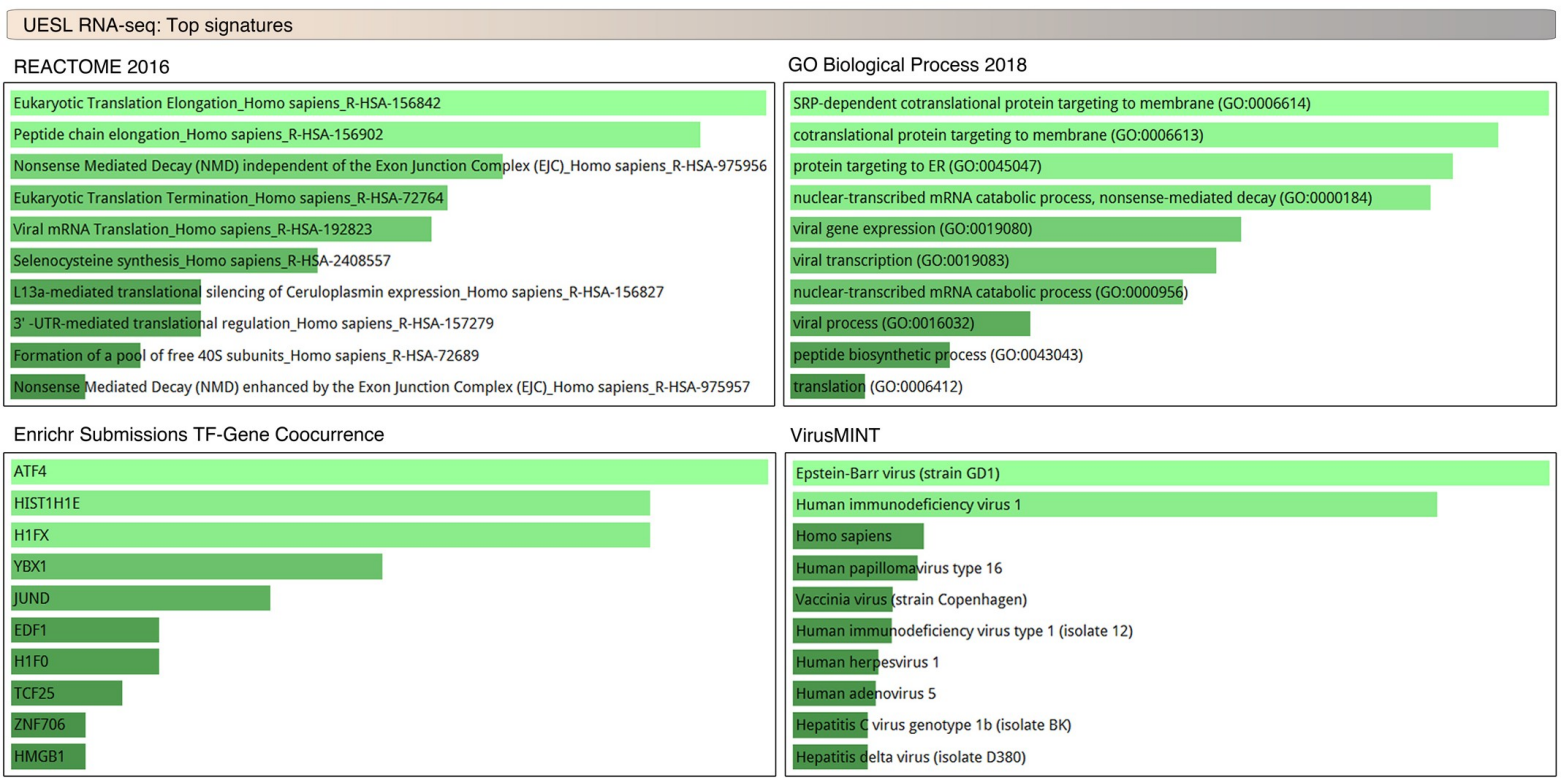
UESL display enriched expression of differentiation and viral response gene pathways. EnrichR analysis of RNA-seq TPM normalized genes of UESL samples. Notably, the most highly enriched gene pathways include transcription factors and epigenetic regulators that have been associated with regulation of cellular differentiation and/or viral response gene sets.

To understand the transcriptional signature of UESL we performed gene set enrichment analysis of the most highly expressed genes across the cohort. Analysis revealed significant enrichment of gene sets associated with transcription factor and epigenetic modulators of cellular differentiation including *ATF4*, *HIST1H1E*, *HIFX*, and *JUND* (Fig 5 and S3 Table), where high expression is associated with a more undifferentiated state [41–44]. Additionally, viral-responsive mRNA transcription and translation-related genes were also significantly enriched in UESL (Fig 5 and S3 Table) and several of the most highly enriched transcriptional factor gene sets such as *ATF4* [45] and *YB-1*[46] can also be induced as a response to viral infection. Considering the well-established role of C19MC in placental physiology regulating trophoblast differentiation and antiviral response [17], our results suggest a causal role of C19MC in the expressional perturbations noted.

## Discussion

In this study, we perform comprehensive molecular analysis of undifferentiated embryonal sarcoma of the liver utilizing a combination of next-generation sequencing and high-density arrays. We find that UESL are characterized by marked aneuploidy, recurrent structural variants near the start site of C19MC leading to pronounced C19MC overexpression, and highly recurrent *TP53* mutation or copy number loss. We further confirm the C19MC fusion event in one of the tumors, which can potentially replace the putative C19MC transcriptional regulatory region with *PEG3/ZIM2* gene, explaining the markedly aberrant transcriptional activity of C19MC in this sample is likely driven by *PEG3/ZIM2* promoter. Resultant hyperexpressed C19MC miRNAs are expressed in a negatively correlated manner to *TP53* and *KRAS* regulatory miRNAs. Expressional analysis revealed that UESL exhibits enrichment of gene sets related to regulators of cellular differentiation, viral response elements, and translation machinery.

Previously reports have documented recurrent chromosomal translocations in the C19MC locus in mesenchymal hamartoma of the liver as well as in several cases of UESL [8]. These prior reports of UESL, however, did not identify that the functional consequence of these fusion events is to lead to extreme overexpression of C19MC. Additionally, while individual C19MC miRNAs have been demonstrated to be overexpressed in MHL [40, 47], to our knowledge a comprehensive evaluation of C19MC miRNA expression in either MHL or UESL has not previously been performed. Furthermore, we find it striking that all UESL tumors in our study that demonstrated C19MC overexpression also had evidence of *TP53* mutation or copy number loss. Given the clinical evidence that some UESL arise from MHL, and that both UESL and MHL share C19MC structural variations, we hypothesize a multistep model for UESL genomic development: C19MC translocation could be the first event leading to a benign tumor, and in some cases *TP53* mutation/loss occurs to lead malignant transformation to UESL (S2 Fig). Notably, both *TP53* alteration and C19MC overexpression may influence common downstream pathways, including K-Ras activation. Our UESL miRNA-seq results with C19MC miRNAs negatively correlating to TP53 and K-Ras regulatory miRNAs support this hypothesis. Further studies, such as confirming C19MC overexpression but lack of *TP53* mutation in MHL, and further understanding of the direct influence of C19MC miRNAs on p53 and K-ras function would be necessary to confirm our proposed model.

Previous reports have implicated C19MC as driver of cell proliferation in several tumor types both benign and malignant [9, 10, 12–14, 48]. Most analogous to our current findings, embryonal tumors with multilayer rosettes (ETMR) are now known to be characterized by C19MC fusion leading to extreme C19MC overexpression [9]. Unlike ETMR, where C19MC fusion is consistently with the same gene partner, *TTYH1*, in UESL C19MC fusions have been described with a variety of partners [8], including the novel *PEG3/ZIM2*-C19MC fusion described in this study.

Interestingly, C19MC is known to play a critical role in cellular differentiation and immunologic (anti-viral) response in placental physiology. Concordantly, we find differentiation and viral response gene sets to be the most highly enriched in UESL. Considering the role of C19MC in antiviral response [49] and our gene expression signature of UESL matching to viral mRNA transcription and translation, it would be of interest to further evaluate for a potential viral etiology in this disease. Alternatively, “viral response” gene signatures may be surrogates for alterations in autophagy because autophagy is critical for C19MC-driven antiviral response [49] and both K-Ras and p53 are tightly linked to autophagy in cancers [50]. Our data on C19MC miRNA negative correlation to *KRAS-* and *TP53*-regulatory miRNAs supports this notion. In this context, presence or absence of functional p53 could render tumor suppressor or oncogenic function of C19MC respectively, as Ras-driven tumors with p53 loss require autophagy for mitochondria function[50]. Further study of the bidirectional regulatory relationship of C19MC miRNAs and *TP53* would be of interest.

Given the increasing interest in C19MC as an oncomir cluster across multiple cancer types and the notable relationship between C19MC overexpression and *TP53* loss/mutation, further study is warranted to examine the oncologic consequences of C19MC overexpression. Given the central importance of C19MC in UESL development, UESL would be well placed as a model system for further studies on this oncomir with potentially wide applicability of findings across cancer types.

## Materials and methods

### Patient tissue samples and processing

Tissue samples were obtained from the Children’s Oncology Group (COG) rare tumor repository. Tumors were classified as an embryonal sarcoma by a sarcoma pathologist at the host institution using standing histological techniques. We initially received extracted nucleic acids or representative tissue samples from 19 patients diagnosed with UESL, including 10 matched tumor-normal pairs and 9 patients with tumor only samples. Matched normal samples were whole blood for 9 patients and adjacent liver tissue for 1 patient. Fresh frozen tissue was utilized for 18 of the tumors, with formalin fixed tissue for the remaining 1 sample. Accompanying patient demographic data and deidentified pathological reports were also reviewed. For a subset of patients with quality concerns from the materials received, we performed post-hoc secondary pathological analysis of histological images from 5 tumor samples of concern. Upon our review, images from 4 of the 5 tumors lacked evidence of any viable tumor, displaying entirely necrosis and/or normal liver. The remaining tumor displayed a mix of necrosis (approximately 30%) with viable sarcoma cells. Given these findings, we excluded these 5 samples from analysis and proceeded with the remaining 14.

All specimens for sequencing were obtained from patients with appropriate consent from their local institutional review board in accordance with the Children’s Oncology Group and the National Cancer Institute. DNA/RNA were extracted from qualifying tumor samples (fresh frozen/FFPE) and matched blood using either AllPrep DNA/RNA Mini Kit (Qiagen #80204) or AllPrep DNA/RNA FFPE Kit (Qiagen #80234) or miRNeasy FFPE Kit (Qiagen #217504) or miRNeasy Mini Kit (Qiagen #217004). The RNA samples were subjected to on-column RNAse free DNAse (Qiagen #79254) digestion as per manufacturer’s protocol. The nucleic acids were quantified using Qubit, and subjected to quality control using Bioanalyzer before proceeding to sequencing.

### Whole exome sequencing

Molecular studies included whole exome sequencing on all tumors and matched normal where available and with nucleic acids of sufficient quantity and quality. Two hundred ng of DNA was used as input into the Agilent SureSelect XT Clinical Research Exome kit, which includes the exon targets of Agilent’s v5 whole-exome kit, with increased coverage at 5000 disease-associated targets. For each DNA sample, a genomic DNA library was constructed according to the manufacturer’s protocol, and the size and quality of the library was evaluated using the Agilent BioAnalyzer. An equimolar amount of library DNA was used for a whole-exome enrichment using the Agilent capture baits and after quantitative PCR library quantitation and QC analysis on the BioAnalyzer. Paired-end 76bp sequencing was performed on Illumina NextSeq 500 instrument. The Burrows-Wheeler Aligner was used to align sequence reads to the reference genome hg19.[51] The Genome Analysis Toolkit was used for insertion/deletion realignment, quality score recalibration, and identification of single nucleotide and insertion/deletion variants.[52] To enrich for somatic mutations, we restricted our analysis to variants that are rare or absent in population databases (MAF <0.01 in 1000 Genomes Project, the NHLBI Exome Sequencing Project, and ExAC database). We additionally utilized annotation by curated databases including COSMIC and the Cancer Gene Census to manually review variants for functional consequence and known status as an oncogene/tumor suppressor gene. We defined putatively pathogenic mutations as those that are hotspot missense mutations or those that are truncating mutations (stopgain, splice site, frameshift) in a characterized tumor suppressor gene.

### RNA sequencing and analysis

RNA samples were processed for RNA-sequencing using the NuGen Ovation Human FFPE RNA-Seq Multiplex System (NuGEN Technologies, San Carlos, CA). Briefly, 100 ng of RNA was used to generate cDNA and a strand-specific library following the manufacturer’s protocol. Quality control steps including BioAnalyzer library assessment and quantitative RT-PCR for library quantification were performed. The libraries were then sequenced the Illumina NextSeq 500 v2 sequencer with 75-base paired-end runs in order to generate approximately 85M million read pairs per sample. Raw sequence reads were mapped to reference genome (hg19) using TopHat2.0. Fusion analysis was done using TopHat 2.0 and DeFuse 0.6. Expression FPKM results were obtained at both gene and transcript level using CuffLinks 2.1. Averages were taken for all TPM normalized genes in UESL samples and the 682 genes that show average expression >50 were subjected to EnrichR analysis [53] to identify the key gene signatures over-represented in UESL. The adjusted p-value 0.0001 was used as cut-off.

### SNP array

SNP arrays were performed on tumor specimens with sufficient DNA quantity and quality using the CytoScan HD array from 250 ng of DNA according to the manufacturer’s protocol (Thermo Fisher). Copy number state and allelic ratio were assessed utilizing the Chromosome analysis suite v3.3.0.139 (Thermo Fisher) and confirmed by manual inspection.

### Targeted panel C19MC sequencing and fusion-breakpoint confirmation

Targeted sequencing of the C19MC breakpoint region was performed utilizing a custom targeted DNA panel (Qiagen, Qiaseq) designed to target the region from chr19:54139995-chr19:54220005 using hg19 coordinates. The design was predicted to cover 96.3% of the target capture region. Tumor DNA underwent library preparation and target enrichment using single primer extension technology according to the manufacturer’s protocol and then subjected to 150bp paired-end sequencing using the Illumina NextSeq 500 platform. Sequencing reads were mapped to hg19 genome using the BWA algorithm. Fusion detection tools including Breakdancer[54, 55], Manta[56], and InFusion[57] were utilized to predict breakpoint events and mapped sequencing data were visualized in IGV.

For a candidate fusion in tumor from patient ID: PATWXD, primers were designed to span the predicted breakpoint. The primer combination that map to C19MC (5’- ATGGTCAGCCTGGGCAGGGTAGC-3’) and ZIM2/PEG3 gene (5’- TCCTTTGCCCGAGGGCTCATGTTG-3’) were used to amplify the fusion sequence, purified using GFX columns (Illustra GFX PCR DNA and Gel Band Purification Kit: GE Healthcare, USA; # 28903470) and subjected to Sanger sequencing using the primers listed above in addition to another set of nested primers which are designed based on IGV. The nested primers are for C19MC, 5’- ACCTTGGTCAATATGGCGAAACCC-3’ and 5’- GATGTTCCTGTTCCCACAATTCTGG-3’. Using the Sanger sequence, the breakpoint was mapped based on hg19 coordinates.

### Small-RNA-seq and analysis of C19MC miRNAs

Fresh frozen UESL and FFPE normal samples were subjected to RNA isolation using miRNeasy mini kit (Qiagen # 217004) and the samples with quality RNAs were subjected to small-RNA-sequencing using the QIAseq miRNA Library Kit according to the manufacturer’s protocol (Qiagen). An average of 17M reads per sample were generated on an Illumina NextSeq 500 instrument using 75 bp single reads, and the raw data were analyzed using the QIAseq miRNA Primary Data Analysis pipeline. Unique molecular identifiers (UMIs) were counted, and miRNA sequences were mapped and counted using the Qiagen software. miRNAs that had cumulative expression above 100 in 7 UESL samples were log transformed and subjected to correlation analysis using R using package ‘corrplot’ 0.84 (was built under R version 3.4.4). The scripts used were, > cor(FileName); > mat <- cor(FileName); > col1 <- colorRampPalette(c("black", "white", "red")); > corrplot(mat, order = "hclust", method = "color", tl.cex = 0.5, col = col1(100)). The positively correlated C19MC miRNA cluster and corresponding negatively correlated miRNA clusters were subjected corrplot using the scripts above with an additional script for significance: > res1 <- cor.mtest(mat, conf.level = .95); > corrplot(mat, order = "hclust", method = "color", tl.cex = 0.5, col = col1(100), p.mat = res1$p, insig = "blank"). Therefore, white or blank represents low or no significant correlation.

UESL miRNA-seq data was used to examine 3p versus 5p mature miRNA expression by calculation of cumulative expression of individual C19MC miRNAs (for all C19MC miRNAs with 5p vs. 3p data available) in the sequenced UESL samples. The cumulative 5p and 3p data for C19MC miRNAs were used to get the 5p to 3p ratio and the miRNAs were classified into 3p dominant if the ratio is ≤0.5, neutral if the ratio is close to 1 and 5p dominant if the ratio is ≥1.5.

## Supporting information

S1 Fig Genome wide copy number variation of UESL. For each tumor, SNP array data are plotted including log2 ratio (top) and allele difference (bottom) to provide an overview of genome-wide CNV. Figures were generated using Chromosome Analysis Suite.
(TIF)

S2 Fig Proposed multistep genomic development of UESL. C19MC overexpression due to chromosomal structural event and *TP53* mutations are the genomic hallmarks of UESL.
(TIF)

S1 Table miRNAs that form C19MC correlatively expressed set-1 and 2 and the list of TP53 and K-Ras regulatory miRNAs that are negatively correlated to C19MC miRNAs. (XLSX)

S2 Table Putatively oncogenic somatic mutations in matched tumor-normal sequencing cohort. (XLSX)

S3 Table EnrichR p-value based ranking of gene sets associated with UESL RNA-seq dataset. (XLSX)
