# Supplementary figures and images for: The genomic landscape of undifferentiated embryonal sarcoma of the liver is typified by C19MC structural rearrangement and overexpression combined with TP53 mutation or loss
Source: PLoS Genet. 2020 Apr 20;16(4):e1008642. doi: 10.1371/journal.pgen.1008642 (PMC7192511; doi:10.1371/journal.pgen.1008642)

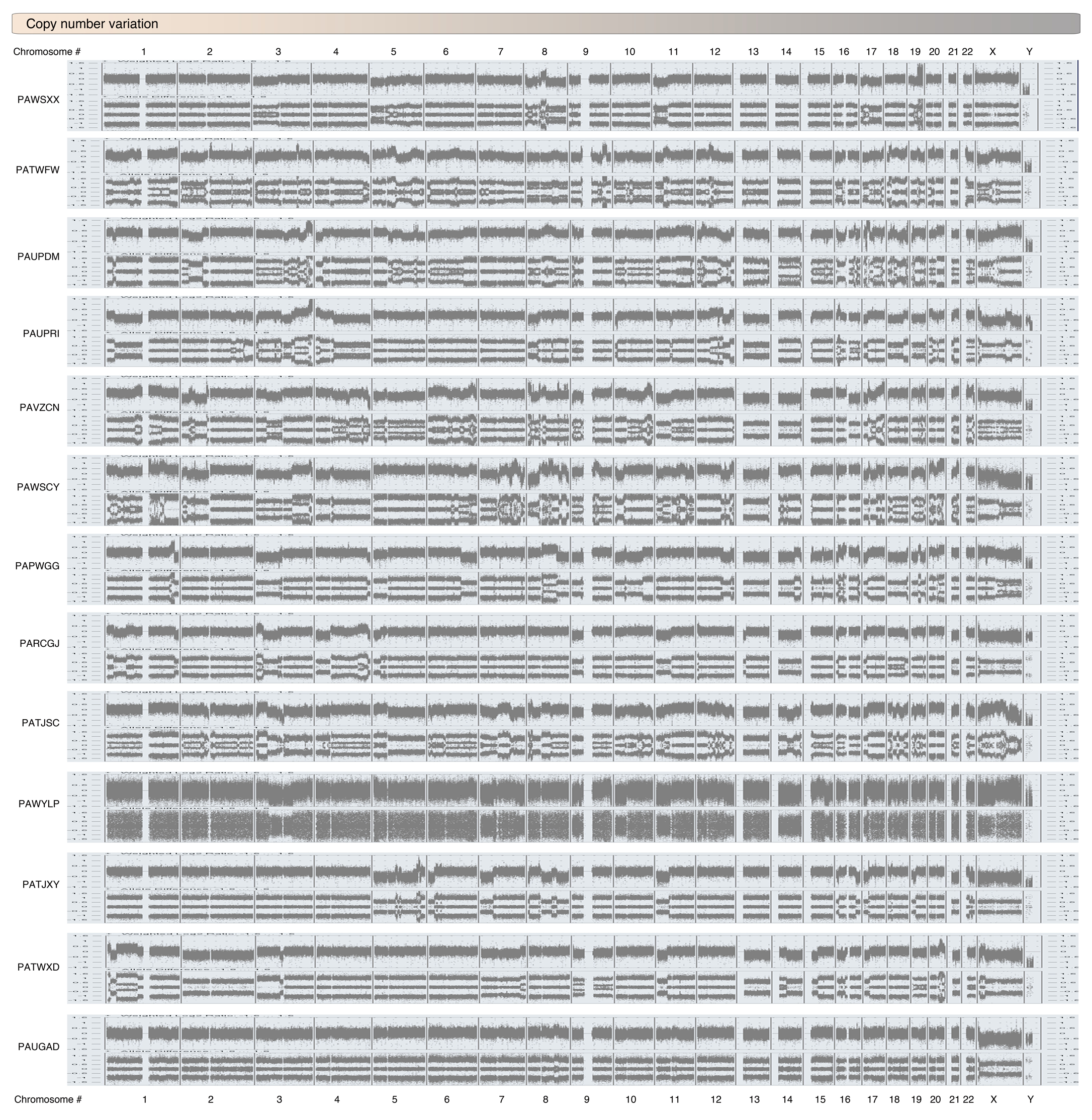

Supplement: S1 Fig — For each tumor, SNP array data are plotted including log2 ratio (top) and allele difference (bottom) to provide an overview of genome-wide CNV. Figures were generated using Chromosome Analysis Suite. (TIF) [file pgen.1008642.s001.tif]

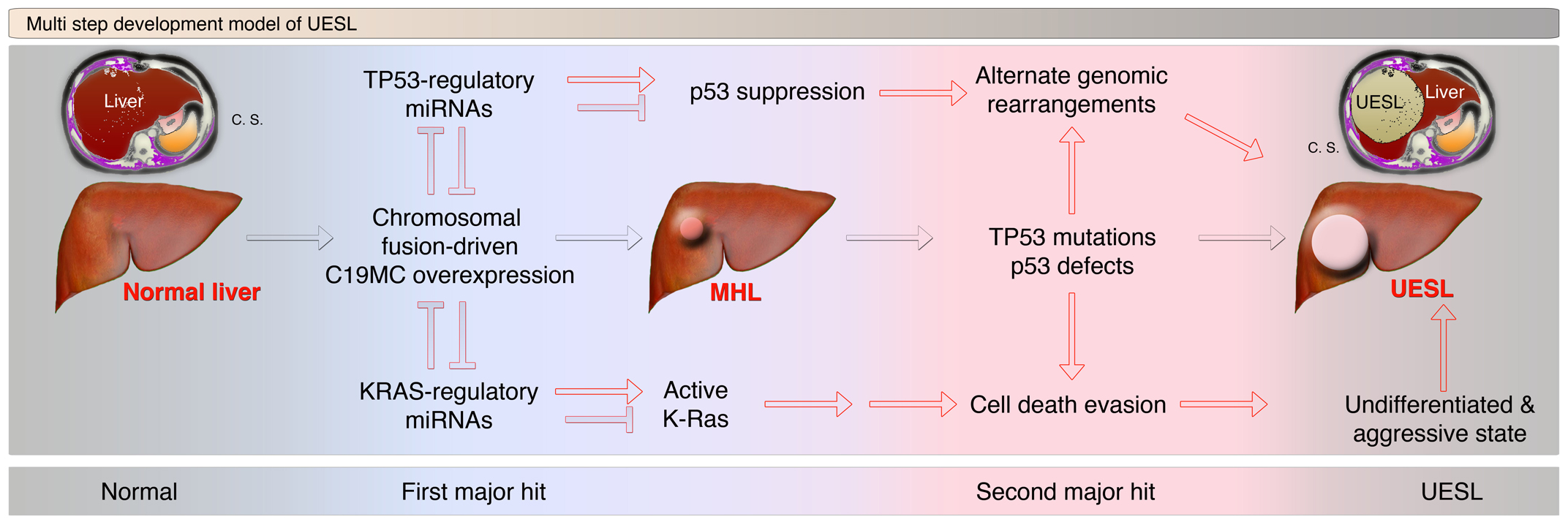

Supplement: S2 Fig — C19MC overexpression due to chromosomal structural event and TP53 mutations are the genomic hallmarks of UESL. (TIF) [file pgen.1008642.s002.tif]
